# Antibacterial activity of methanol extracts of the leaves of three medicinal plants against selected bacteria isolated from wounds of lymphoedema patients

**DOI:** 10.1186/s12906-020-03183-0

**Published:** 2021-01-03

**Authors:** Dereje Nigussie, Gail Davey, Belete Adefris Legesse, Abebaw Fekadu, Eyasu Makonnen

**Affiliations:** 1 Centre for Innovative Drug Development and Therapeutic Trials for Africa (CDT-Africa), College of Health Sciences, Addis Ababa University, P.O. Box 9086, Addis Ababa, Ethiopia; 2 Centre for Global Health Research, Brighton and Sussex Medical School, University of Sussex, Brighton, BN1 9PX UK; 3 School of Public Health, Addis Ababa University, P.O. Box 9086, Addis Ababa, Ethiopia; 4 Department of Pharmacology and Clinical Pharmacy, College of Health Sciences, Addis Ababa University, Addis Ababa, Ethiopia

**Keywords:** Lymphoedema, Wound infection, Bacteria, Medicinal plants, Ethiopia

## Abstract

**Background:**

Patients with lymphoedema are at high risk of getting bacterial and fungal wound infections leading to acute inflammatory episodes associated with cellulitis and erysipelas. In Ethiopia, wound infections are traditionally treated with medicinal plants.

**Methods:**

Agar well diffusion and colorimetric microdilution methods were used to determine the antibacterial activity of methanol extracts of the three medicinal plants against *Staphylococcus aureus, Streptococcus pyogenes, Escherichia coli, Klebsiella pneumoniae, Pseudomonas aeruginosa, Shewanella alage*, methicillin-resistant *S. aureus* ATCC®43300TM, *Staphylococcus aureus* ATCC25923, *Escherichia coli* ATCC25922, *Klebsiella pneumoniae* ATCC700603, and *Pseudomonas aeruginosa* ATCC37853.

**Results:**

The methanol extract of *L. inermis* leaves showed high activity against all tested bacterial species, which was comparable to the standard drugs. Similarly, the extracts of *A. indica* showed activity against all tested species though at higher concentrations, and higher activity was recorded against *Streptococcus pyogenes* isolates at all concentrations. However, the extract of *A. aspera* showed the lowest activity against all tested species except *Streptococcus pyogenes* isolates. The lowest minimum inhibitory concentration (MIC) was recorded with the extract of *L. inermis* against *E. coli* isolate and *S. aureus* ATCC 25923.

**Conclusion:**

Methanol extracts of *L. inermis, A. indica*, and *A. aspera* leaves exhibited antimicrobial activity against selected bacterial isolates involved in wound infections, of which the methanol extracts of *L. inermis* exhibited the highest activity. The results of the present study support the traditional use of plants against microbial infections, which could potentially be exploited for the treatment of wound infections associated with lymphoedema.

## Background

Wound infections are usually associated with normal flora, bacteria from the environment or hospital-acquired infections [1]. Microorganisms infect soft tissue when the skin surface is compromised in some way. Patients with lymphoedema are at high risk of wound infection because of loss of skin integrity [2], resulting in ingress of microorganisms. Impaired clearance of microorganisms from the infected area due to impaired lymphatic system results in recurrent infections [3]. Patients with secondary lymphoedema are predisposed to cellulitis [4].

Acute inflammatory episodes associated with cellulitis and erysipelas are common complications of wounds in lymphoedema patients, and most infections are caused by group A, C, or G streptococci and *Staphylococcus aureus* bacteria [2]. Microorganisms known to cause chronic wound infection and cellulitis in lymphoedematous limbs include *Streptococci species*, *Staphylococci species*, *Pseudomonas species*, and *Bacteroides species* [5]. Fungal infections are also common due to the moisture produced between the skin folds, resulting in skin breakdown, which in turn leads to infection in the macerated regions. Lymphangitis is an inflammation of the lymphatic system due to bacterial infection after invasion through skin wounds or abrasions [6].

Folk medicine provides an important and unexplored resource for the discovery and development of potential new medicines against microbial infections to decrease the emergence of resistance and adverse drug reactions. Furthermore, the use of medicinal plants provides opportunities for developing countries as they may be more affordable, accessible and available [7].

Ethnobotanical studies carried out in Ethiopia reported that endemic plants have been used by traditional healers for a range of ailments, including open wound infections. However, the scientific evidence available regarding the antibacterial activity of traditionally used endemic plants against bacterial pathogens involved in wound infections of lymphoedematous limbs is limited [8].

This study, therefore, aimed to test the antibacterial activity of the leaf methanol extracts of *Lawsonia inermis*, *Azadirachta indica*, and *Achyranthus aspera* against selected bacteria isolated from the wounds of patients with lymphoedema and against standard ATCCs.

## Methods

### Plant material collection

The leaves of *Lawsonia inermis* (Henna) were collected from Laga Gandisa (approximately 9^0^ 32′ 59″ N and 41^0^ 28′ 31″ E), 53 km from Dire Dawa, Ethiopia. The leaves of *Azadirachta indica* (Neem) were collected from Kurare Goti (approximately 10^0^ 7′ 15″ N and 38^0^ 9′ 13″ E), 209 km northwest of Addis Ababa on the way to Dejen Town, Ethiopia. The leaves of *Achyranthes aspera* (Telenge) were collected from the Nile Gorge (approximately 10^0^ 7′51″N, and 38^0^9′19″ E), 210 km northwest of Addis Ababa on the way to Dejen, Ethiopia. No access permit was required from Ethiopian Biodiversity institute for the collection of these plants. Collection of all plant materials was carried out in consultation with a botanist from the Ethiopian Public Health Institute, local people, and traditional healers in the areas. Plant materials were authenticated by a botanist and specimens were archived at the Herbarium of Ethiopian Public Health Institute with voucher numbers of MG-012/05 for *L. inermis*, NA10 for *A. aspera*, and DG-18 for *A. indica.*

### Bacterial strains

The bacterial strains for this experiment were isolated from the wounds of lymphoedema patients from East Wollega Zone. Standard reference bacteria were obtained from the National Referral Bacteriology and Mycology Laboratory, Ethiopian Public Health Institute. Clinical isolates of *Staphylococcus aureus, Streptococcus pyogenes, Escherichia coli, Klebsiella pneumoniae, Pseudomonas aeruginosa,* and *Shewanella alage* were used. Reference bacteria including MRSA *Staphylococcus aureus* ATCC®43300™*, Staphylococcus aureus* ATCC25923*, Escherichia coli* ATCC25922*, Klebsiella pneumoniae* ATCC700603 and *Pseudomonas aeruginosa* ATCC37853 were also used.

### Swab sample collection and processing

Wounds were cleaned with sterile normal saline and wound swabs and discharge were obtained from all study participants aseptically using a sterile moistened cotton swab. Swabs were then immersed in a container of Amies transport medium with charcoal (Bio mark Laboratories, Pune, India). All samples were transported on ice to the Ethiopian Public Health Institute, National Referral Bacteriology and Mycology Laboratory (Ethiopian National Accreditation Office accredited and ranked as Five Star by the American Society for Microbiology) where all laboratory tests were conducted. Swabs were used to inoculate MacConkey agar (Becton Dickinson and Company, Cockeysville, MD, USA), blood agar and mannitol salt agar (both from HiMedia Laboratories, Mumbai, India) and incubated aerobically at 37 °C, and 5% CO2 for 24 h. After 24 h, plates without growth were incubated further for up to 48 h.

Growth of micro-organisms was identified by examining colony morphology followed by biochemical identification using the automated VITEK® 2 COMPACT Microbial Detection System (bioMerieux, Marcy l’Etoile, France).

### Extraction and preparation of plant materials

The extraction of each plant material was done following methods previously used with slight modifications [9]. Each plant material powder was successively extracted with three organic solvents in order of increasing polarity (petroleum ether ➔ethyl acetate➔methanol➔aqueous). Three hundred grams of each powder was soaked in 1.5 l of petroleum ether separately and kept on a VWR DS 500 orbital shaker for 72 h. The extracts were filtered using WhatmanNo1 filter paper. The residue was further extracted twice with fresh petroleum ether. Then, all the filtrates were mixed. The resulting residues were air-dried and further extracted with ethyl acetate, methanol, and sterile water using the procedure employed for petroleum ether extraction. Organic solvents were then removed from the extracts using rotavapor and the extracts were kept for several days in a water bath (40 °C). After complete drying, the yield of each extract was measured separately, and the extracts were stored at 4^0^c until used for further study. The dried extracts of *A. aspera*, *L. inermis* and *A. indica* were dissolved in 10% DMSO and kept at 4^0^c until used for the experiments.

### Preliminary phytochemical screening of the extracts

Phytochemical analysis of the methanol extract of *A. aspera, L. inermis* and *A. indica* leaves was performed using standard procedures to determine the active constituents present in the extracts. Tests for alkaloids, saponins, phenols, tannins, anthraquinones, terpenoids, flavonoids and steroids were performed following the methods developed before [10].

#### Test for alkaloids

Extracts from plants were dissolved in HCl and filtered for the following tests.

- **Mayer’s Test**: Filtrates were treated with Mayer’s reagent (Potassium Mercuric Iodide). Yellow precipitation indicates the presence of alkaloids in the extracts.
- **Dragendroff’s Test**: Filtrates were treated with Dragendroff’s reagent (solution of Potassium Bismuth Iodide). Red precipitation indicates the presence of alkaloids in the extracts.

#### Test for saponins (foam test)

Extract was shaken with 2 ml of water. If foam produced persists for 10 min, it indicates the presence of saponins.

#### Phenol test

0.5 G crude extracts was treated with a few drops of 2% FeCl_3_ bluish green or black colouration indicated presence of phenolic compound

#### Test for tannins (ferric chloride test)

Each plant extract was stirred with 1 ml of distilled water, after filtered, ferric chloride reagent added to the filtrate. A blue-black, green, or blue-green precipitate indicates the presence of tannins.

#### Test for anthraquinones

**C**hloroform was added to the extracts and shaken for 5 min. The extract was filtered and shaken with an equal volume of 100% ammonia solution. A pink, violet or red colour in the ammoniacal layer (lower layer) indicated the presence of free anthraquinones.

#### Test for terpenoids

Each extract was dissolved in chloroform, then 3 ml of concentrated sulfuric acid was added carefully and examined: reddish brown coloration indicates the presence of terpenoid.

#### Test for steroids

Chloroform 10 ml was added to 2 ml of all three plant extracts. To these extracts 1 ml of acetic anhydride was added and then 2 ml of concentrated sulphuric acid was added along the sides of the test tube. Colour formation at the junction is noted. The appearance of blue green colour indicates the presence of steroids.

#### Test for flavonoids (alkaline reagent test)

Extracts were treated with drops of sodium hydroxide solution. Formation of intense yellow colour, which becomes colourless on addition of dilute acid, shows presence of flavonoids.

### Bacterial culture and inoculum preparation

Fresh cultures of bacteria were prepared from frozen stock, streaked on Mueller Hinton agar (MHA) plates and incubated for 24 h at 37 °C in an incubator. After- 18-24 h of incubation, a single colony of microorganisms was picked and inoculated into 3 mL sterile saline solution. The saline tube was then vortexed to create a uniform solution, and turbidity was adjusted to 0.5 McFarland standard (10^8^ CFU/mL).

### Antibacterial activity assays

#### The agar well diffusion assay

The Kirby-Bauer technique was used to determine the antibacterial activity of the extracts [11]. A total of 11 bacteria strains were used for this test. Mueller Hinton agar (pH 7.2 & 4 mm depth) plates were inoculated with test organisms (prepared in a sterile saline tube) by streaking the loop in a back-and-forth motion to ensure an even distribution of inoculum. MHA with 5% sheep blood was used for *Streptococcus pyogenes*. A circular 6 mm diameter well was punched aseptically with a sterile cork borer. Then, a volume of 100 μL methanol extracts of *A. aspera*, *L. inermis* and *A. indica* leaves (at concentrations of 100 mg/mL, 200 mg/mL, and 400 mg/mL) were dispensed into the wells. Similarly, 5% Di-methyl-sulfoxide (DMSO) was dispensed into the control well, and reference antibiotic discs were placed on the surface of the plate and incubated for 24 h at 37 °C. For *Streptococcus pyogenes,* a carbon dioxide incubator was used for incubation. Each experiment was done three times.

#### Microdilution method

The minimum inhibitory concentration (MIC) and minimum bactericidal concentration (MBC) of the extracts were determined using the p-iodonitrotetrazolium chloride (INT) colorimetric assay method [12]. The test was done according to the recommendations of the Clinical Laboratory Standard Institute [13]. Bacteria were sub-cultured on Mueller Hinton agar (pH 7.2) and incubated at 37 °C for 24 h. Bacterial colonies were inoculated into a sterile saline solution and used before 30 min. The bacterial suspension was evenly mixed and diluted to meet the turbidity of 0.5 McFarland standard (1 × 10^8^ CFU/mL). Further dilution was performed to obtain the final concentration of inoculum (5 × 10^5^ CFU/mL) in each well. A stock solution of plant extracts was prepared in DMSO. Serial dilutions of the working solution were prepared by diluting the extract solution in sterile Mueller Hinton Broth. The final concentration of DMSO in the solution was less than 2.8% in the solution. The test was performed in a sterile 96-well plate. Methanol extracts of *A. aspera*, *L. inermis*, and *A. indica* leaves were tested in triplicate in one plate for each bacterium. Mueller Hinton Broth (100 μL) was dispensed to all wells. A working solution of extracts (100 μL) and solvent controls (MH broth and 2.8% DMSO) were dispensed into their respective wells. Serial dilutions were performed from columns one to nine, and 50 μL of excess medium was discarded from column nine. The last column served as a blank containing only broth. Columns 10 and 11 served as negative controls, which only contained medium and bacterial suspension, and media DMSO and bacteria, respectively. 50 μL of test bacteria were added to each well except for the last row, which served as a blank. The concentration of plant extracts ranged from 0.78 mg/mL to 200 mg/mL. The plates were sealed and incubated for 24 h at 37 °C. After 24 h incubation, 40 μL (0.2 mg/mL) p-iodonitrotetrazolium chloride (INT) was added to all wells and incubated again at 37 °C for 30 min. The MIC of the samples was detected after 30 min of incubation. Viable bacteria reduced the yellow dye to pink. MIC was defined as the sample concentration that prevented the colour change of the medium and exhibited complete inhibition of microbial growth. The MBC was determined by adding 50 μL aliquots from the wells that did not show growth after incubation for the MIC test to 150 μL broth in the well plate, and incubated for 48 h at 37 °C. Then, MBC was observed as the lowest concentration of extracts which did not produce a colour change after the addition of INT as mentioned above.

### Statistical analysis

Statistical Package for Social Science (SPSS) version 20 was used for descriptive analyses, such as frequencies and means. Statistical differences in the mean zones of inhibition for individual bacteria and differences in the susceptibility of the test microorganisms were analysed using ANOVA followed by Tukey’s post hoc at a significance level of *P* < 0.05. MIC was analysed using descriptive statistics.

## Results

### Plant extract yield and properties

The percent yields of the methanol extracts of *A. aspera*, *L. inermis* and *A. indica* and their properties are given in Table 1. Maximum yield was obtained from *L. inermis* (15.9%)*,* followed by *A. aspera* (14.7%) and *A. indica* (7.9%).

**Table 1:** Extraction yield of the plants in methanol

Plant name with parts	Appearance	Consistency	Yield (% w/w)
*Lawsonia inermis* (leaves)	Brown	Semisolid	15.9
*Achyranthes aspera* (leaves)	Dark green	Semisolid	14.7
*Azadirachta indica* (leaves)	Grey	Powder	7.9

### Preliminary phytochemical screening of the extracts

The methanol extracts of *L. inermis, A. aspera* and *A. indica* leaves tested positive for alkaloids, terpenoids, phenolic compounds, tannins and steroids tests. Furthermore, *L. inermis* contained anthraquinones, whereas *A. indica* contained saponins and flavonoids. However, *L. inermis* did not contain flavonoids or saponins, and flavonoids were absent from *A. aspera*. *A. indica* was negative for the anthraquinones test (Table 2).

**Table 2:** Preliminary phytochemical screening for secondary metabolites

S/N	Secondary metabolites	*L. inermis*	*A. aspera*	*A. indica*
1	Alkaloids	+	+	+
2	Terpenoids	+	+	+
3	Saponins	+	–	+
4	Flavonoids	–	–	+
5	Phenols	+	+	+
6	Tannins	+	+	+
7	Anthraquinones	+	–	–
8	Steroids	+	+	+

+ = present, − = absent

### Antibacterial activity

The antibacterial activities of the methanol extracts of *L. inermis, A. aspera* and *A. indica* leaves were tested against microorganisms isolated from the wounds of patients with lymphoedema and standard ATCCs. In vitro antibacterial activity was tested in the presence or absence of a zone of inhibition in diameter, the minimum inhibitory concentration (MIC) and minimum bactericidal concentration (MBC) in comparison with the reference antibacterial drugs.

Generally, it was observed that bacterial growth inhibition increased as the concentration of the extracts increased. Pairwise comparison of ANOVA was used to test the variability in susceptibility of the microorganisms toward the extracts (*p* < 0.05). No significant difference was observed in terms of susceptibility between *K. pneumoniae* isolates and ATCC (*p* = 0.91), *S. algae* isolates and *P. aeruginosa* ATCC (*p* = 0.74), *E. coli* isolates and *K. pneumoniae* ATCC (*p* = 0.89), *S. aureus* isolates and MRSA ATCC (*p* = 1.0), or *S. aureus* isolates and *E. coli* ATCC (*p* = 1.0). There was a significant difference in the zone of inhibition between *L. inermis* and the other two plant extracts, *A. aspera* and *A. indica*. However, no significant growth inhibition difference was detected between *A. indica* and *A. aspera* (*p* = 0.55).

**Fig. 1:**
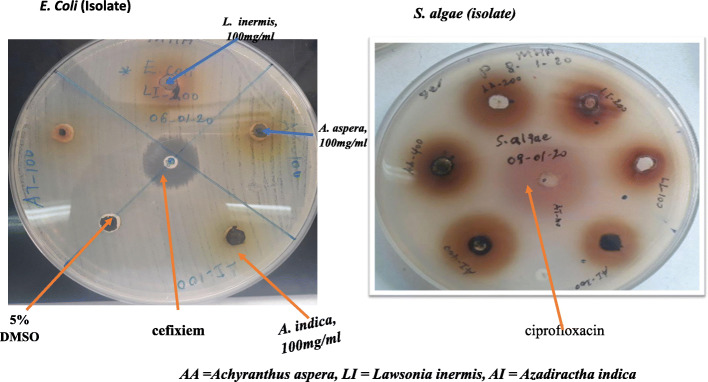
Zone of inhibition of extracts against *E. coli* and *S. algae* isolates

**Fig. 2:**
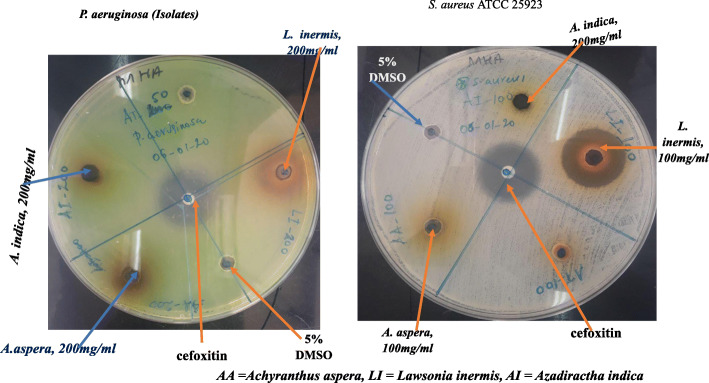
Zone of inhibition of extracts against *P. aeruginosa* and *S. aureus* isolates

The *Streptococcus pyogenes* isolate showed the highest susceptibility to all the extracts at all concentrations compared with the standard drugs*. K. pneumoniae ATCC700603, K. pneumoniae* isolate*s* and *P. aeruginosa* isolates showed low levels of susceptibility against all extracts (Figs. 1 and 2).

All three concentrations (100, 200, and 400 mg/mL) of *L. inermis* showed significant activity against all tested bacteria species, which was comparable to the standard drugs. The highest zone of inhibition was recorded by *L. inermis* against all tested species except *K. pneumonia* ATCC700603*, P. aeruginosa* isolates and *K. pneumonia* isolates. *L. insermis* showed exceptional activity against *E. coli* isolates*, S. aureus* ATCC 25923*,* and MRSA ATCC® 43300™, which was comparable to the conventional drug cefoxitin (Table 3).

**Table 3:** Mean inhibition zone of bacterial growth (mm) for the leaves of methanol extracts of *L. inermis, A. aspera and A. indica* leaves

Plants	Conc. (mg/mL)	Zone of inhibition (mm) (Mean ± SD)										
		*S. aureus*		*E. coli*		*P. aeruginosa*		*K. pneumoniae*		MRSA	*S. pyogenes*	*Shewanella algae*
		ATCC	Isolate	ATCC	Isolate	ATCC	Isolate	ATCC	Isolate	ATCC	Isolate	Isolate
LI	100	33.0 ± 1.0	15.5 ± 0.5	15.1 ± 0.7	8.1 ± 0.4	20.5 ± 0.5	12.5 ± 0.5	8.2 ± 0.3	7.6 ± 0.5	15.1 ± 0.7	21.0 ± 1.0	20.5 ± 0.5
	200	31.0 ± 1.0	17.5 ± 0.5	19.3 0.6	10.3 ± 0.8	21.2 ± 0.7	13.8 ± 0.8	9.6 ± 0.5	8.8 ± 0.7	19.3 ± 0.6	24.5 ± 0.5	21.2 ± 1.1
	400	33.0 ± 1.0	21.3 ± 1.5	21.2 ± 0.3	12.1 ± 0.6	21.5 ± 0.9	15.6 ± 0.5	10.5 ± 0.9	10.8 ± 0.8	21.2 ± 0.3	25.9 ± 0.9	21.9 ± 1.0
AA	100	7.1 ± 0.6	6.3 ± 0.6	6.4 ± 0.5	6.8 ± 0.8	6.4 ± 0.4	6.2 ± 0.3	6.4 ± 0.4	6.0 ± 0.0	6.6 ± 0.6	10.5 ± 0.9	6.5 ± 0.5
	200	9.1 ± 0.4	6.5 ± 0.5	6.6 ± 0.6	7.4 ± 0.5	6.5 ± 0.5	6.6 ± 0.5	6.8 ± 0.7	6.5 ± 0.5	7.5 ± 0.6	12.9 ± 1.0	6.6 ± 0.7
	400	6.3 ± 0.4	7.0 ± 1.0	7.5 ± 0.6	7.6 ± 0.5	6.7 ± 0.6	7.0 ± 1.0	7.2 ± 1.0	6.5 ± 0.5	7.4 ± 0.4	13.3 ± 0.6	6.8 ± 0.8
AI	100	6.4 ± 0.4	6.2 ± 0.3	6.3 ± 0.4	6.6 ± 0.5	6.4 ± 0.4	6.4 ± 0.4	6.4 ± 0.5	6.1 ± 0.2	6.3 ± 0.4	16.7 ± 0.6	6.3 ± 0.6
	200	6.7 ± 0.3	6.7 ± 0.6	6.5 ± 0.5	7.4 ± 0.4	6.8 ± 0.7	6.8 ± 0.7	6.6 ± 0.6	6.1 ± 0.2	6.5 ± 0.5	18.3 ± 0.6	7.4 ± 0.5
	400	7.4 ± 0.4	8.2 ± 0.7	7.5 ± 0.7	6.3 ± 0.3	7.8 ± 0.2	7.5 ± 0.5	8.5 ± 0.5	7.4 ± 0.5	6.4 ± 0.5	21.0 ± 1.0	9.5 ± 0.5
5 μg cefoxitin		27.0 ± 0.0	27.0 ± 0.0	24.0 ± 0.0	24.0 ± 0.0	29.0 ± 0.0	29.0 ± 0.0	24.0 ± 0.0	24.0 ± 0.0	15.0 ± 0.0	–	–
5 μg ciprofloxacin		–	–	–	–	–	–	–	–	–	–	24.0 ± 0.0
10 μg penicillin		–	–	–	–	–	–	–	–	–	24.0 ± 0.0	–
5% DMSO		NI	NI	NI	NI	NI	NI	NI	NI	NI	NI	NI

Values are triplicate and represented as mean ± SD. *AI Azadirachta indica, LI Lawsoni ainermis*, *AA Achranthes aspera, NI* No inhibition

*A. indica* extract was shown to have activity against all tested species at high concentrations, and high activity was recorded against *Streptococcus pyogenes* isolates at all concentrations (100, 200, and 400 mg/mL). However, 100 mg/mL and 200 mg/mL concentrations showed low activity against all tested strains. *A. aspera* showed the lowest activity against all tested species, except against *Streptococcus pyogenes* isolate (10.5 ± 0.9 to 13.3 ± 0.6 mm ZI) compared with the reference drug cefoxitin (15–24 mm) (Table 3). There were significant differences in the mean zone of inhibition between the different concentrations of *L. inermis*, *A. aspera* and *A. indica* (*p* < 0.05).

Among the strains, *S. aureus*, *E. coli*, *P. aeruginosa*, and *K. pneumonia* isolates were less susceptible to *L. inermis* than the standard ATCCs. Similarly, multi-drug resistant *S. aureus* (MRSA) was less susceptible to all tested extracts compared to *S. aureus* isolate and standard ATCC. All the references used in the test showed the highest activity against their respective tested species. The mean inhibition zones of triplicate experiments for the three different concentrations of extracts (100, 200 and 400 mg/mL) are summarized in Table 3.

The methanol extracts of the three plant leaf extracts showed different levels of MIC against all tested bacteria. There was no inhibition of growth of bacteria in the negative controls (medium and bacterial suspension, and media DMSO and bacteria). The colorimetric broth microdilution assay showed that the methanol extract of *L. inermis* inhibited the growth of eleven tested microorganisms within the concentration ranges of 1.5 ± 1.4 to 12.5 ± 0.0 mg/mL. Whereas, the minimum bactericidal concentration (MBC) ranged from 12.00 ± 0.0 to 83.3 ± 28.9 among the strains.

The MICs were recorded for *L. inermis* against *E. coli* isolate (1.5 ± 1.4 mg/mL) and *S. aureus* ATCC 25923 (3.1 ± 0.0 mg/mL), and the lowest MBC against *E. coli* isolate (12.00 ± 0.0 mg/mL). The highest MIC value of *L. inermis* was against *K. pneumoniae* ATCC700603 and *E. coli* ATCC 25922 which was 12.0 ± 0.0 mg/mL for both (Table 4).

**Table 4:** The mean values of MIC and MBC for the methanol extracts of *Lawsonia inermis*, *Achyranthes aspera* and *Azadirachta indica* leaves

S/N	Bacteria	MIC (mg/mL)			MBC (mg/mL)		
		LI	AI	AA	LI	AI	AA
1	*S. aureus* ATCC 25923	3.1 ± 0.0	33.3 ± 14.4	50.0 ± 0.0	25.0 ± 0.0	200.0 ± 0.0	200.0 ± 0.0
2	MRSA *S. aureus* ATCC® 43300™	4.2 ± 2.0	33.3 ± 14.4	42.0 ± 14.4	50.0 ± 0.0	200.0 ± 0.0	> 200.00
3	*E. coli* ATCC 25922	12.5 ± 0.0	83.3 ± 29.0	66.7 ± 28.9	25.0 ± 0.0	200.0 ± 0.0	200.0 ± 0.0
4	*P. aeruginosa* ATCC27853	4.2 ± 1.8	50.0 ± 0.0	200.0 ± 0.0	18.8 ± 10.8	200.0 ± 0.0	200.0 ± 0.0
5	*K. pneumoniae* ATCC700603	12.5 ± 0.0	41.7 ± 14.4	50.0 ± 0.0	83.3 ± 28.9	100.0 ± 0.0	> 200.00
6	*E. coli* isolate	1.5 ± 1.4	83.3 ± 28.9	100.0 ± 0.0	12.00 ± 0.0	100.00 ± 0.0	200.0 ± 0.0
7	*K. pneumoniae* isolate	7.3 ± 4.8	25.0 ± 0.0	50.0 ± 0.0	50.0 ± 0.0	100.0 ± 0.0	> 200
8	*P. aeruginosa* isolate	12.5 ± 0.0	100.0 ± 0.0	166.7 ± 57.7	66.7 ± 28.9	200.0 ± 0.0	> 200
9	*Shewanella algae* isolate	6.25 ± 0.0	100.0 ± 0.0	200.0 ± 0.0	83.3 ± 28.9	100.0 ± 0.0	> 200
10	*S. aureus* isolate	6.25 ± 0.0	25.0 ± 0.0	50.0 ± 0.0	25.0 ± 0.0	50.0 ± 0.0	100.0 ± 0.0
11	*S. pyogenes* isolate	6.25 ± 0.0	33.3 ± 14.4	83.3 ± 28.9	41.7 ± 14.4	36.7 ± 23	100.0 ± 0.0

Values are triplicate and represented as mean ± SD. *MIC* Minimum inhibitory concentration, *MBC* Minimum bactericidal concentration, *LI Lawsonia inermis, AI Azadirachta indica*, *AA Achyranthes aspera*

Similarly, the MICs of *A. indica* ranged from 25.0 ± 0.0 mg/mL to 100.0 ± 0.0 mg/mL among the tested strains, and MBC ranges from 36.7 ± 23 to 200.0 ± 0.0 mg/mL. The lowest MIC of *A. indica* was observed against *S. aureus* and *K. pneumonia* isolates, and the highest values were against *P. aeruginosa* and S. *algae* isolates (Table 4). The lowest MBC of *A. indica* was observed against *S. pyogenes* isolate (36.7 ± 23 mg/mL) (Table 3.10). Similarly, the MICs of methanol extracts of *A. aspera* ranged from 50.0 ± 0.0–200.0 ± 0.0 mg/mL. The minimum bactericidal concentration for the three plant extracts was ≥200.0 mg/mL, except for *S. aureus* isolate and *S. pyogenes* isolate which was 100.0 ± 0.0 mg/mL for both (Table 4).

## Discussion

Natural products contain a range of lead compounds which may enable the development of novel antimicrobial agents as conventional antimicrobial drugs become ineffective due to emergence of resistance. The secondary metabolites present in a medicinal plant may have different modes of antimicrobial action which help combat the emergence of resistance [14].

Assessing the antibacterial activity of herbal medicines for their potential use in treatment of skin and wound infections has great importance. The antibacterial activity observed in the present study suggests that among the plant extracts tested are some that could be used for the management of wound infections in patients with lymphoedema.

Qualitative tests for secondary metabolites in methanol extracts of *L. inermis* leaves revealed the presence of alkaloids, terpenoids, saponins, phenols, tannins, anthraquinones and steroids which may be responsible for the antibacterial activity demonstrated. Which is in agreement The metabolites documented are in line with previous findings [15] with the exception of the absence of flavonoids. *L. inermis* is a source of unique and valuable natural compounds that have been considered for a wide range of disease conditions as well as cosmetics [16].

The methanol extract of *L. inermis* leaves had significant activity against all tested bacteria. Among the tested strains, *S. aureus* ATCC 25923, MRSA ATCC®43300TM, *E. coli* ATCC 25922, *E. coli*, and *Streptococcus pyogenes* isolates were the most susceptible, with a zone of inhibition comparable to cefoxitin and penicillin at all tested concentrations. This finding supports the work of Manivannan et al. [17], Kannahi et al. [18], and Badoni et al. [19]. ß-asarones, the active constituents found in the leaves, roots, and rhizomes of the *L. inermis,* were responsible for all antimicrobial activities [20].

The quinones present in *L. inermis* (henna) were found to possess high activity against all microorganisms [17]. In the methanol extract of *L. Inermis* leaves*,* alkaloids, anthraquinones, and saponins have been reported to have antibacterial activity, and the high activity against most microorganisms may be due to a single or combined effect of these secondary metabolites [21].

*Azadirachta indica* is one of the most useful medicinal plant, whose plant parts have been used as traditional medicine with proven antiseptic, antiviral, antipyretic, anti-inflammatory, antiulcer and antifungal properties [22]. Our study showed that the methanol extract of *A. indica* leaves contained alkaloids, terpenoids, saponins, flavonoids, phenols, tannins, and steroids, which is in agreement with previous reports [22]. Phytochemical constituents such as flavonoids and saponins could be responsible for the anti-inflammatory, antimicrobial, antioxidant, and antiviral activity of the plant [22].

*A. indica extract* was found to have moderate activity against all tested strains except *E. coli* isolate (6.3 ± 0.3 mm ZI) and MRSA (6.4 ± 0.5 mm ZI) at 400 mg/mL. In comparison with the reference drugs, the highest activity of *A. indica* extract was recorded against *Streptococcus pyogenes* isolate (21.0 ± 1.0 mm ZI), followed by *Shewanella algae* (9.0 ± 0.5 mm ZI) at 400 mg/mL. Clinical isolates of *E. coli*, *P. aeruginosa*, and *K. pneumonia* strains were found to be less susceptible than the standard ATCCs at the tested concentrations. *A. indica* extract at concentrations of 100 mg/mL and 200 mg showed low activity against the tested strains, except for *Streptococcus pyogenes* and *S. algae* (Fig. 2).

*S. aureus* isolates were more susceptible than the standard ATCC. Previous studies showed that the methanol extract of *A. indica* (neem) had high activity against standard and clinical isolated strains of *P. aeruginosa* [23]. Another study indicated that ethanol extracts of *A. indica* (neem) leaf exhibited antibacterial activity against *E. coli*, *K. pneumoniae*, *Proteus mirabilis*, *S. aureus*, *P. aeruginosa*, and *Enterococcus faecalis* at 100, 50, and 25 mg/mL [24].

*Achyranthes aspera* locally known as “Telegne” is traditionally used in Ethiopia for treatment of a range of wound infections [25]. It was reported to have anti-bacterial, anti-inflammatory, analgesic, and antipyretic activities [26]. Previous studies on methanol extracts of *A. aspera* (leaves) showed secondary metabolites such as alkaloids, terpenoids, phenols and tannins which might be responsible for the pharmacological activities of the plant extract [27].

In this study, the methanol extract of *A. aspera* leaves showed high antibacterial activity against *Streptococcus pyogenes* at 400 mg/mL, and low activity against the other strains at tested concentrations. Next to *Streptococcus pyogenes*, *S. aureus* ATCC was found to be more susceptible than the clinical isolate. Except for the *Streptococcus pyogenes* strain, this finding agrees with the report of Taye et al [28], in which gram-positive bacteria were more susceptible than gram-negatives to the plant extracts, perhaps due to the nature of their cell walls. Gram-negatives have phospholipid membranes carrying structural lipopolysaccharide components that makes their cell wall impermeable to some antimicrobial substances [29].

The minimum inhibitory concentration (MIC) is defined as the lowest concentration of antimicrobial agent that inhibited the visible growth of microorganisms after overnight incubation. The MBC is complementary to the MIC. It demonstrates the lowest level of antimicrobial agent that results in microbial death after sub culturing the organism in an antibiotic-free media [30]. The MIC is used to evaluate the antimicrobial effectiveness of new compounds or extracts by measuring the effect of decreasing the antimicrobial concentration. Antimicrobials with lower MIC are considered to be more effective.

The MIC values obtained from the present study indicated that the methanol extract of *L. inermis* leaves was more potent against *E. coli* isolate and *S. aureus* ATCC 25923, which agrees with the initial antimicrobial screening test results (agar well diffusion test). Strong antibacterial activity was also observed against *S. aureus* ATCC 25923, MRSA ATCC® 43300TM, and *P. aeruginosa* ATCC27853 at low concentrations of *L. inermis* extract. The results of our study agree with those of previous report from Jordan [31]. The differences in bacterial susceptibility could be due to variations in intrinsic tolerance of microorganisms, or the physico-chemical properties of phytochemicals present in the crude extracts of the plant materials [31].

The MBC/MIC ratio was determined for *L. inermis* extract to determine whether the extract was bactericidal or bacteriostatic at the tested concentrations. The MBC/MIC ratio greater than 4 is usually considered to be a bacteriostatic effect; whereas values less than 4 show bactericidal effects [32]. Accordingly, *L. inermis* extract was shown to have bactericidal effects against *E. coli* ATCC 25922, *P. aeruginosa* ATCC27853, *E. coli* isolate, *K. pneumonia* isolate, *S. aureus* isolate and *Streptococcus pyogenes* isolate; but bacteriostatic activity against *S. aureus* ATCC 25923, MRSA ATCC® 43300TM, *P. aeruginosa* ATCC27853, *K. pneumonia* ATCC700603, *P. aeruginosa* isolate and *S. algae* isolate. Generally, the activity is considered to be high when MIC is less than10 μg/mL, moderate when MIC is between 10 and100 μg/mL and low when MIC is greater than 100 μg/m [33].

*A. indica* and *A. aspera* extracts with MICs ranging from 25.0 ± 0.0 mg/mL to 100.0 ± 0.0 mg/mL, and 50.0 ± 0.0 to 200.0 ± 0.0 mg/mL, respectively, had moderate to low activity against the tested bacterial strains. Even though *A. indica* exhibited moderate to low activity against the tested strains, it showed bactericidal activity against all tested strains, with MBC values ≥200 mg/mL against all tested strains except *S. aureus* and *Streptococcus pyogenes* isolates.

The presence of bioactive phytochemical compounds such as alkaloids, flavonoids, tannins, phenols, steroids, and terpenoids in all three tested plant extracts accounted (either individually or in combination) for the broad-spectrum antimicrobial activities observed in this study, which is in agreement with the findings of previous studies [34, 35].

Possible modes of antibacterial action of some of the secondary metabolites could be described as follows: Tannins may act by inactivating microbial adhesins, enzymes and cell envelope transport proteins [34]; flavonoids by altering the cell membranes of microbes and inhibiting energy metabolism and synthesis of nucleic acids [36], alkaloids by disrupting the cell wall and /or inhibiting Deoxyribonucleic acid (DNA) synthesis (Ref?); anthraquinones by increasing the levels of superoxide anions and/or singlet oxygen molecules [37], and diterpenes and phenolic compounds by disrupting microbial cell membranes [38].

## Conclusion

The methanol extracts of *L. inermis*, *A. indica* and *A. aspera* leaves exhibited antimicrobial activity against selected bacterial isolates involved in lymphoedema-associated wound infections and standard ATCCs including methicillin resistant *S. Aureus*. *L. inermis* extract demonstrated high activity and had bactericidal effects against most of the tested bacterial strains. However, *A. indica* and *A. aspera* extracts showed low to moderate activity against most tested strains at 400 mg/mL. These findings support the traditional claim that the three medicinal plants have antibacterial activity in wound infections. Further investigations, however, need to be carried out before recommending their use.

## Data Availability

The datasets during and/or analysed during the current study available from the corresponding author on reasonable request.

## Abbreviations

ANOVA: One-way analysis of variance
ATCC: American Type Culture Collection
CC_50_: Minimum dose that is toxic to 50% of cells
CFU: Colony forming unit
CI: Confidence interval
CLSI: The Clinical and Laboratory Standards Institute
DMSO: Di-methyl-sulfoxide
DNA: Ribonucleic acid
mg/mL: milli gram per ml
MHA: Mueller Hinton agar
MHB: Muller Hinton broth
MIC: Minimum inhibitory concentration
IC_50_: Half minimum inhibitory concertation
MD: Mean difference
MDR: Multiple drug resistance
PBS: Phosphate buffered saline
MRSA: Methicillin resistance *Staphylococcus aureus*
SD: Standard deviation
SPSS: Statistical package for social science
WHO: World Health Organization
ZI: Zone of inhibition

## References

[1] Jamal BS, Mohammed IA (2019). Bacterial isolates from wound infections and their antibiotic susceptibility pattern in Kassala teaching hospital, Sudan. Am J Microbiol Res.

[2] Keeley V, Riches K (2009). Cellulitis treatment for people with lymphoedema : UK audit. J Lymphoedema.

[3] Fife CE, Farrow W, Hebert AA, Armer NC, Stewart BR, Cormier JN (2017). Skin and wound care in lymphedema patients : A taxonomy , primer , and literature review. Adv Skin Wound Care.

[4] Ayman A, Grada TJP (2017). Lymphedema pathophysiology and clinical manifestations. Am Acad Dermatology, Inc.

[5] Eagle M (2007). Understanding cellulitis of the lower limb. Wound Essent.

[6] Park SI, Yang EJ, Kim DK, Jeong HJ, Kim GC, Sim YJ (2016). Prevalence and epidemiological factors involved in cellulitis in korean patients with lymphedema. Ann Rehabil Med.

[7] Ali NAA, Ju W (2001). Screening of Yemeni medicinal plants for antibacterial and cytotoxic activities. J Ethnopharmacol.

[8] Mummed B, Abraha A, Feyera T, Nigusse A, Assefa S (2018). *In Vitro* antibacterial activity of selected medicinal plants in the traditional treatment of skin and wound infections in eastern Ethiopia. Biomed Res Int.

[9] Uthayarasa K, Pathmanathan K, Jeyadevan JP, Jeyaseelan EC (2010). Antibacterial activity and qualitative phytochemical analysis of medicinal plant extracts obtained by sequential extraction method. Int J Integr Biol.

[10] Pandey A, Tripathi S, Pandey CA (2014). Concept of standardization, extraction and pre phytochemical screening strategies for herbal drug. J Pharmacogn Phytochem JPP.

[11] Hudzicki J. Kirby-Bauer disk diffusion susceptibility test protocol. American Society for Microbiology; 2016: 3-23.

[12] Kuete V, Betrandteponno R, Mbaveng AT, Tapondjou LA, Meyer JJM, Barboni L (2012). Antibacterial activities of the extracts , fractions and compounds from *Dioscorea bulbifera*. BMC Complement Altern Med.

[13] Clinical and Laboratory Standards Institute (2011). Performance Standards for Antimicrobial Susceptibility Testing; Twenty-First Informational Supplement. CLSI document M100-S21.

[14] Tadesse B, Yinebeb T, Ketema B (2016). Antibacterial activity of selected medicinal plants used in South-Western Ethiopia. African J Microbiol Res..

[15] Usman RA, Rabiu U (2006). Antimicrobial activity of *Lawsonia inermis* ( henna ) extracts. Bayero J Pure Appl Sci.

[16] Khaliq FA, Raza M, Hassan SU, Iqbal J, Aslam A, Aun M, et al. Formulation, characterization and evaluation of *in vivo* wound healing potential of Lawsone ointment. Am J Adv Drug Deliv. 2018;06(01):61-8.

[17] Manivannan R, Aeganathan R, Prabakaran K (2015). Anti-microbial and anti-inflammatory flavonoid constituents from the leaves of Lawsonia inermis. J Phytopharm.

[18] Kannahi M, Nadu T (2013). Antimicrobial activity of *Lawsonia inermis* leaf extracts against some human pathogens. Int J Curr Microbiol App Sci..

[19] Badoni R, Kumar D, Combrinck S, Cartwright-jones C, Viljoen A (2014). *Lawsonia inermis* L. ( henna ): Ethnobotanical , phytochemical and pharmacological aspects. J Ethnopharmacol.

[20] Kamal M, Jawaid T. Pharmacological activities of Lawsonia inermis Linn. |A review. Int J Biomed Res. 2011;1(2).

[21] Dahake PR, Naik P, Pusad M (2015). Study on antimicrobial potential and preliminary phytochemical screening of *Lawsonia inermis* Linn. Int J Pharm Sci Res.

[22] Galeane MC, Martins CHG, Massuco J, Bauab TM, Sacramento LVS (2017). Phytochemical screening of *Azadirachta indica* A . Juss for antimicrobial activity. African J Microbiol Res..

[23] Rasool M, Malik A, Arooj M, Alam MZ, Alam Q, Asif M (2017). Evaluation of antimicrobial activity of ethanolic extracts of *Azadirachta indica* and *Psidium guajava* against clinically important bacteria at varying pH and temperature. Biomed Res- India.

[24] Mohammed HA, Omer AFA (2015). Antibacterial activity of *Azadirachta indica* ( Neem ) leaf extract against bacterial pathogens in Sudan. Am J Res Commun.

[25] Edwin S, Jarald EE, Deb L, Jain A, Kinger H, Dutt KR (2009). Wound healing and antioxidant activity of *Achyranthes aspera*. Pharm Biol.

[26] Srivastav S, Singh P, Mishra G, Jha KK, Khosa RL (2011). *Achyranthes aspera*- An important medicinal plant : A review. J Nat Prod Plant Resour.

[27] Elumalai EK, Chandrasekaran N, Thirumalai T, Sivakumar C, Therasa SV, David E (2009). *Achyranthes Aspera* leaf extracts inhibited fungal growth. Int J PharmTech Res.

[28] Taye B, Giday M, Animut A, Seid J (2011). Antibacterial activities of selected medicinal plants in traditional treatment of human wounds in Ethiopia. Asian Pac J Trop Biomed.

[29] Leach LC (2015). Structural elucidation of some antimicrobial constituents from the leaf latex of aloe. BMC Complement Altern Med.

[30] Owuama CI (2017). Determination of minimum inhibitory concentration ( MIC ) and minimum bactericidal concentration ( MBC ) using a novel dilution tube method. African J Microbiol Res.

[31] Kouadri F (2018). *In vitro* antibacterial and antifungal activities of the saudi *Lawsonia inermis* extracts against some nosocomial infection pathogens. J Pure Appl Microbiol.

[32] Venkateswarulu TC, Srirama K, Mikkili I, Nazneen Bobby M, Dulla JB, Alugunulla VN (2019). Estimation of minimum inhibitory concentration (MIC) and minimum bactericidal concentration (MBC) of antimicrobial peptides of *Saccharomyces boulardii* against selected pathogenic strains. Karbala Int J Mod Sci.

[33] Bianco ÉM, Krug JL, Zimath PL, Kroger A, Paganelli CJ, Boeder AM (2015). Antimicrobial (including antimollicutes), antioxidant and anticholinesterase activities of Brazilian and Spanish marine organisms – evaluation of extracts and pure compounds. Brazilian J Pharmacogn.

[34] Begashaw B, Mishra B, Tsegaw A, Shewamene Z (2017). Methanol leaves extract *Hibiscus micranthus* Linn exhibited antibacterial and wound healing activities. BMC Complement Altern Med.

[35] Wolde T, Bizuayehu B, Hailemariam T, Tiruha K (2016). Phytochemical analysis and antimicrobial activity of *Hagenia abyssinica*. Indian J Pharm Pharmacol.

[36] Gupta PD, Birdi TJ (2017). Development of botanicals to combat antibiotic resistance. J Ayurveda Integr Med.

[37] Malmir M, Serrano R, Silva O, Mendez-Vilas A (2017). Anthraquinones as potential antimicrobial agents-A review. Antimicrobial research: Novel bioknowledge and educational programs. Formatex.

[38] Tamokou JDD, Mbaveng AT, Kuete V (2017). Antimicrobial activities of African medicinal spices and vegetables.

